# Foundation years: a time of reflection

**DOI:** 10.1007/s40037-015-0223-7

**Published:** 2015-10-19

**Authors:** Elliott Yann Ah-kee, Aamir Asif Khan

**Affiliations:** 1 Monklands Hospital, Monkscourt Ave, Airdrie, North Lanarkshire ML6 0JS UK; 2 Glasgow Royal Infirmary, 84 Castle Street, Glasgow, G4 0SF UK

To the Editor,

Reflection is considered a key element of professional development. Junior doctors have an obligation to demonstrate reflection on experience, to learn from practice, and eventually institute these changes into their practice. In the UK, they are expected to maintain a professional development portfolio by recording reflections and learning needs for good medical practice [1]. As junior doctors with an interest in medical education, we believe that there are still many barriers to effective reflection. More resources and organizational change for effective reflection is needed; and furthermore, we need more research to assess the efficacy of specific strategies to enhance reflection and its impact on clinical practice.

Despite the emphasis on reflective practice, there is a lack of understanding for the effective application of reflection in a transformative approach to clinical practice. Moreover, reflective practice can often be perceived as hollow, particularly if done for appraisals or work-based assessments. For junior doctors, reflection has almost become a routine ‘tick-box’ exercise with the incentive to progress through their training. Abiding by this professional requirement has usurped its intended educational goal. Previous studies have shown that many medical students and doctors feel that reflection has become a ritualistic process that does not achieve its intended purpose [2].

We believe that structured training programmes can overcome these aforementioned barriers by (i) conveying an understanding of core principles of reflective practice; (ii) instilling an appreciation of its impact on professional development and learning; and (iii) imparting metacognitive skills to junior doctors and challenging their thought processes via feedback in a supportive environment.

Productive reflection is a developmental process that requires adequate training of supervisors and facilitators. Potential changes imply a large investment in resources. Organizational and cultural changes are also needed to create a safe environment to have assumptions challenged. One holistic solution would be to integrate reflective practice into undergraduate training, allowing individuals sufficient training in positive influences of reflection when beginning their careers.

In a systematic review of reflective practice in the health professions, Mann et al. [3]. found that none of the 29 identified papers were randomized controlled studies. Moreover, there was a lack of significant evidence of improvement in clinical practice via reflection. However, the review showed that reflection enabled a deeper approach to learning, in that learners could process complex situations and integrate new learning to existing skills and knowledge.

Whilst not isolated to junior doctors, reflective evidence can often be overlooked when progressing through one’s career, especially when only in rotations for 4–6 months. Reflection is undoubtedly essential in the effective development of the modern clinician, therefore formal training should be provided in aiding doctors to improve their reflective skills. Further randomized controlled studies are warranted to assess the efficacy of strategies to enhance reflection and its impact on clinical practice. In conclusion, we believe that engaging junior doctors with the accurate process of reflective practice will benefit both patient care and medical education profoundly.

## Disclosures

The authors declare no conflict of interest.
